# Monitoring the myosin crossbridge cycle in contracting muscle: steps towards ‘Muscle—the Movie’

**DOI:** 10.1007/s10974-019-09543-9

**Published:** 2019-07-20

**Authors:** Felicity Eakins, Carlo Knupp, John M. Squire

**Affiliations:** 1 Faculty of Medicine, Imperial College London, Exhibition Road, London, SW7 2AZ UK; 2 School of Optometry and Vision Sciences, Cardiff University, Cardiff, CF10 3NB UK; 3 Muscle Contraction Group, School of Physiology, Pharmacology and Neuroscience, University of Bristol, Bristol, BS8 1TD UK

**Keywords:** Time-resolved X-ray diffraction from muscle, Myosin head organisation, Muscle lattice disorder, Weak-binding myosin head state, Interacting heads motif, Muscle M3 meridional peak

## Abstract

Some vertebrate muscles (e.g. those in bony fish) have a simple lattice A-band which is so well ordered that low-angle X-ray diffraction patterns are sampled in a simple way amenable to crystallographic techniques. Time-resolved X-ray diffraction through the contractile cycle should provide a movie of the molecular movements involved in muscle contraction. Generation of ‘Muscle—The Movie’ was suggested in the 1990s and since then efforts have been made to work out how to achieve it. Here we discuss how a movie can be generated, we discuss the problems and opportunities, and present some new observations. Low angle X-ray diffraction patterns from bony fish muscles show myosin layer lines that are well sampled on row-lines expected from the simple hexagonal A-band lattice. The 1st, 2nd and 3rd myosin layer lines at d-spacings of around 42.9 nm, 21.5 nm and 14.3 nm respectively, get weaker in patterns from active muscle, but there is a well-sampled intensity remnant along the layer lines. We show here that the pattern from the tetanus plateau is not a residual resting pattern from fibres that have not been fully activated, but is a different well-sampled pattern showing the presence of a second, myosin-centred, arrangement of crossbridges within the active crossbridge population. We also show that the meridional M3 peak from active muscle has two components of different radial widths consistent with (i) active myosin-centred (probably weak-binding) heads giving a narrow peak and (ii) heads on actin in strong states giving a broad peak.

## Introduction

**Fig. 1:**
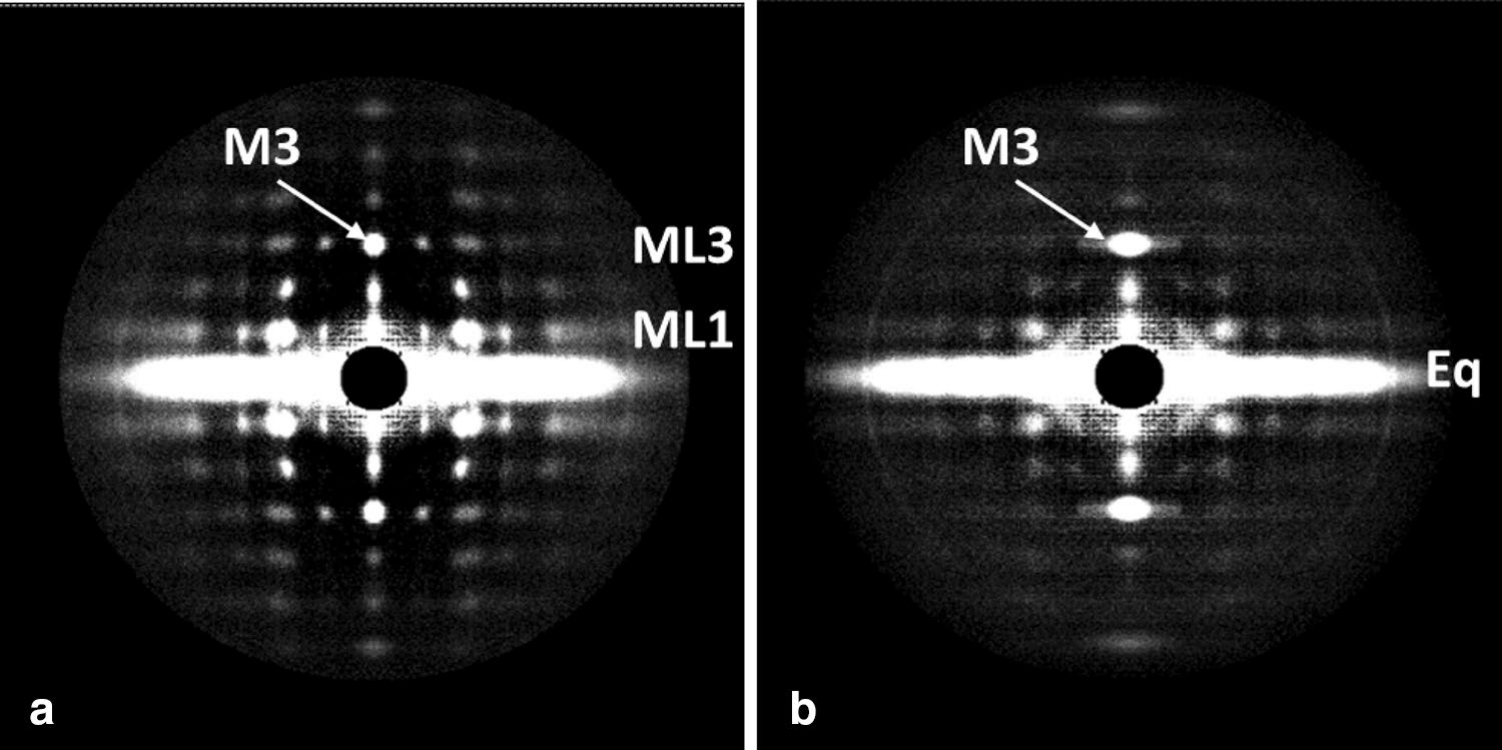
Low-angle X-ray diffraction patterns from bony fish fin muscle either relaxed (**a**) or fully active (**b**). The fibre axis is vertical and the length of the line focus on Daresbury beamline 16.1 is in the vertical direction to give optimal definition along the (horizontal) layer lines. Reflections highlighted are the M3 meridional reflection at 14.3 nm on the ML3 layer line, the equator (Eq) and the ML1 1st myosin layer line at an axial spacing of 43 nm

Of all the vertebrate muscles being studied by structural methods, it is muscles like those of bony fish that promise to provide the most complete evidence on the cross-bridge cycle. This is because bony fish are a good example of animals with a systematically ordered simple lattice arrangement of myosin filaments (Luther and Squire 1980; Luther et al. 1981, 1996; Harford and Squire 1986). In principle, the beautifully sampled low-angle X-ray diffraction pattern from fish muscle (Fig. 1), recorded throughout a tetanic contraction (Eakins et al. 2016), can be solved using conventional crystallographic analysis methods to provide a ‘movie’ of cross-bridge behaviour throughout the contractile cycle (Harford and Squire 1992, 1997; Squire et al. 1994; Squire and Knupp 2005, 2017). In practice, there remain a few technical demands which still need to be overcome. However, there are features of the pattern that can already be analysed in detail, as was done for the equatorial intensities in the study by Eakins et al. (2016). Here we present in a qualitative way some aspects of the layer-line pattern from active bony fish muscle and discuss how changes in the layer-lines and meridional peaks that occur through the contractile cycle can be evaluated in a more analytical way.

All muscle myosin filaments appear to have a common axial spacing of about 14.3 to 14.5 nm between ‘crowns’ of myosin heads (Huxley and Brown 1967; Reedy 1968; Squire 1972, 1973, 1981, 2009). In vertebrate striated muscles, where in relaxed frog muscle the crown repeat is 14.34 nm (Huxley and Brown 1967), and in relaxed bony fish where it is 14.32 nm (Harford and Squire 1986), the myosin filaments have threefold rotational symmetry with three pairs of myosin heads in each crown (Squire 1972; Kensler and Stewart 1983; Zoghbi et al. 2008; Al-Khayat et al. 2013). The axial repeat along the filaments is about 43 nm. Meridional reflections in X-ray diffraction patterns from resting frog and bony fish muscles appear as orders of 43 nm, with the 3rd order, M3, at around 14.3 nm and the 6th, M6, at around 7.2 nm relatively strong (Fig. 1a). Weaker meridional peaks, sometimes referred to as the forbidden meridional reflections (Huxley and Brown 1967; Harford and Squire 1986), occur at M1, M2, M4, M5 etc. (Fig. 1a). Because, in fully active isometric muscle, the myosin heads label the neighbouring actin filaments at axial positions close to the myosin crowns, diffraction patterns from contracting frog and bony fish muscles also show strong M3 and M6 peaks (Huxley and Brown 1967; Squire and Harford 1988; Knupp et al. 2009). The M3 peak is also evident in patterns from rigor muscle, where all the heads are thought to be labelling actin filaments (Yagi 1996; Lovell et al. 1981; Cooke and Franks 1980; Eakins et al. 2018). The rigor labelling pattern has been analysed (Harford and Squire 1994; Yagi 1996; Eakins et al. 2018) and this shows how actin-attached myosin heads can still produce a strong M3 reflection.

Linari et al. (2000) showed that active isometric frog muscle gave an M3 intensity (I_M3_) which scales directly with sarcomere length (S), reducing towards zero at S = 3.6 μm (non-overlap). The active M3 reflection, therefore, appears to come mainly from myosin heads and solely from those heads overlapping the actin filaments; the heads not overlapped by actin must be disordered and must contribute little.

The M3 reflection has been the subject of intense study, including elegant experiments recording M3 interference changes from active muscle (e.g. Huxley et al. 1982; Lombardi et al. 1995; Irving et al. 1992, 2000; Dobbie et al. 1998; Linari et al. 2000; Bagni et al. 2001; Piazzesi et al. 2002; Reconditi et al. 2004; Ferenczi et al. 2005; Brunello et al. 2006; Griffiths et al. 2006; Huxley et al. 2006a, b). These experiments were originally interpreted in terms of the active M3 coming solely from heads attached to actin, with the M3 intensity depending on the position of the lever arm of the actin-attached heads. Then a population of detached heads was also introduced, but in those papers the lever arm tilt was retained as an important factor in determining M3 intensity. More recently, Knupp et al. (2009), questioned this interpretation and suggested that the ‘detached’ population, by which was meant heads either properly detached or heads weakly and non-stereospecifically-attached to actin, is in fact the major population and is very much more highly axially ordered than previously suggested. At the same time they showed that changes in the lever arm orientation contribute little to the observed meridional diffraction pattern.

One of the early observations on active frog muscle, which has been substantiated in more recent studies, is that the M3 meridional peak appears to be broader when the muscle is activated in an isometric contraction. The broadening has been attributed to disordering of the axial alignment of adjacent myosin filaments when the muscle is activated and possibly to the effects of the slightly lower regularity of the actin filaments, since at least part of the ‘active’ M3 comes from heads labelling actin (Huxley et al. 1982; Knupp et al. 2009). However, when the total intensity in the M3 peak is corrected for this increase in peak width in the way described by Huxley et al. (1982), and justified analytically by Eakins et al. (2016), the total integrated M3 intensity increases in patterns from active muscle relative to relaxed. Various estimates for the ratio *I*_*M3*_*active/I*_*M3*_*relaxed*, all corrected in the same way, fall in the range 1.4 to 2.0 (Huxley et al. 1982; Bordas et al. 1993; Juanhuix et al. 2001; Brunello et al. 2006; Griffiths et al. 2006; Oshima et al. 2007). Clearly it is important that any model of the crossbridge cycle can explain this apparent increase in intensity from resting to active muscle.

Here we also analyse the intensity distribution along the myosin ML1 to ML3 layer lines (at orders of 43 nm) and show that the distribution of intensity in patterns from active muscle is not just a reduced version of the resting layer line intensity. The active ML1 to ML3 layer lines come from a different head conformation that is part of the active cycle. We also analyse the contributions to the M3 X-ray reflection and ML3 layer line (Fig. 1) in resting muscle and any changes that might occur when the muscle is activated. We conclude that, in addition to a minority of heads strongly bound to actin in active muscle (previously estimated to be roughly 30%; e.g. Eakins et al. 2016), a significant ordered population of heads are ‘detached’ heads which are probably in a rapid equilibrium between truly detached and a weakly actin-bound (non-stereospecifically-attached) state, with their lever arms relatively perpendicular to the fibre axis. This is consistent with our earlier estimate of myosin head configurations based on other data (Knupp et al. 2009; Eakins et al. 2016). Finally, our aim here is to show how the changing structure of the myosin heads through the contractile cycle can, in principle, be followed by time-resolved X-ray diffraction analysis. We do not show a movie, but we discuss what is still needed to produce ‘Muscle—The Movie’ (Squire et al. 1994).

## Materials and methods

### Muscle dissection, activation and control

Details of the bony fish muscle preparation were given in Eakins et al. (2016). In brief, flatfish (Plaice, *Pleuronectes platessa*) from the London University Marine Biological Station (U.M.B.S.), Isle of Cumbrae, Scotland or from Aquarium Technologies Ltd. (Weymouth, UK), were kept alive in tanks of re-circulating sea water at 5–7 °C for up to 1 week. Whole fin muscles were dissected as in the protocol in the Supplementary Material of Eakins et al. (2016), and set up in custom built, cooled, specimen chambers containing James and Johnston (1998) Ringers solution.

Muscles held at 7–8 °C were activated electrically using platinum wires along each side of (but not touching) the muscle, and stimulation was at 17 V, 140 Hz, with a pulse width of 0.08 ms. Optimal sarcomere rest length was 2.3 µm. Diffraction patterns were recorded using the RAPID detector (Lewis et al. 1997) on beamline 16.1 at the CCLRC Daresbury Synchrotron Radiation Source (SRS). The camera length was 4 to 4.5 m. The timing protocol had the initial resting phase recorded for 100 ms, the tetanus rising phase sampled at 1 ms intervals, the tension plateau exposed for 100 ms and the relaxation phase recorded at 4 ms intervals. Muscle length was controlled by a laser diffraction feedback system. The positions of the two 1^st^ order peaks from the sarcomere diffraction pattern were monitored and changes were fed back to a motor length-control system. Active tension was monitored throughout (see Eakins et al. 2016).

The focus of beam line 16.1 was a short horizontal line, about 1 mm by 0.5 mm. For the present study the muscles were horizontal to optimise detail across the meridian and along the layer lines. For this reason fine sampling along the meridian from axial interference functions could not be seen. The actin layer lines were also axially smeared because of this specimen and beam configuration and they appeared very weak compared to the sampled myosin layer lines.

### Diffraction pattern analysis

**Fig. 2:**
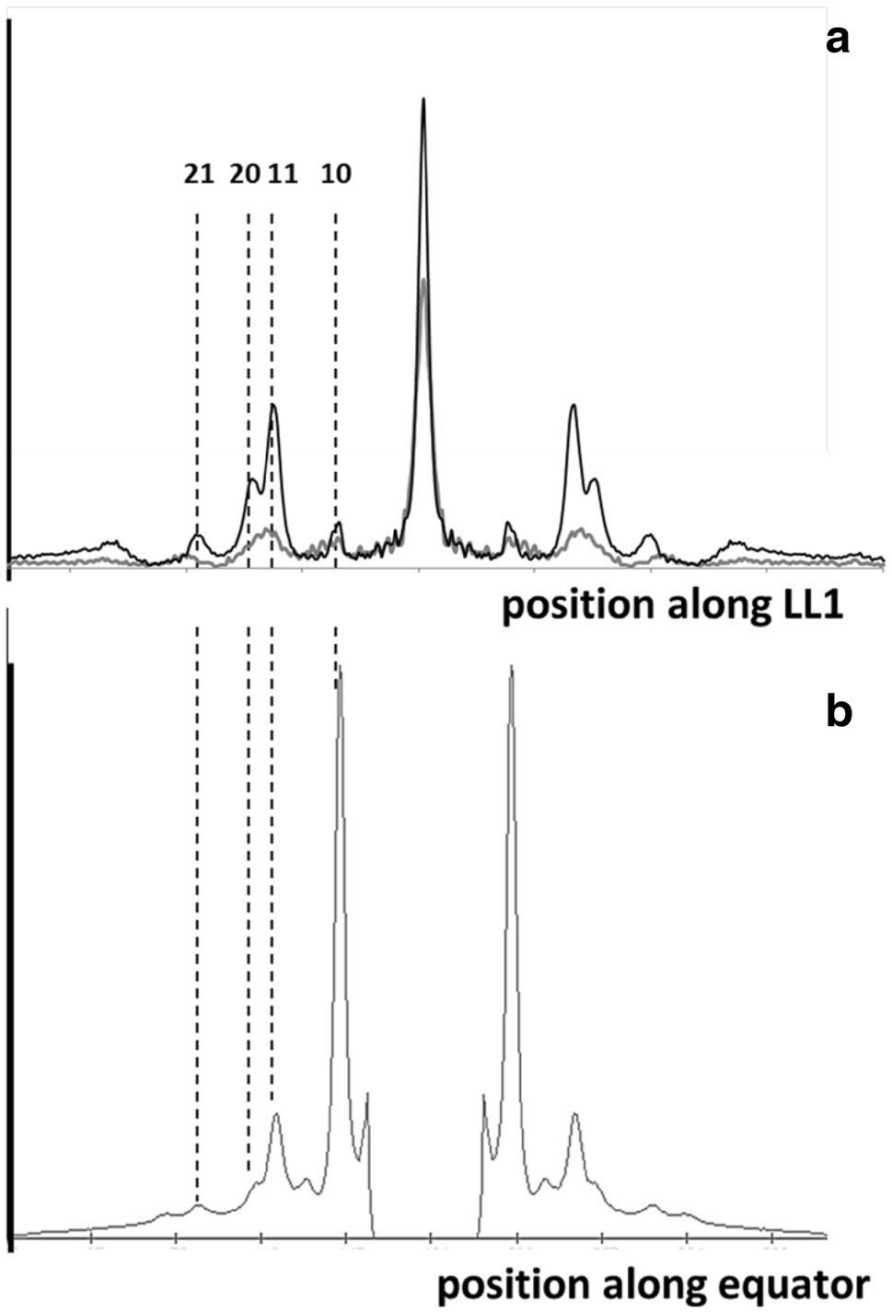
Intensity profiles: **a** from the first myosin layer line ML1 in patterns from resting (dark line) and contracting (grey line) bony fish muscle taken from patterns such as those in Fig. 1, and **b** along the equator of the same, relaxed, diffraction pattern as in **a**. The positions of the 10, 11, 20 and 21 sampling peaks (row lines) are indicated by dashed vertical lines. The intensity scales in **a** and **b** are not the same; the equator is relatively very strong. The patterns in **a** show a general reduction of intensity when the muscle is activated, but the sampling peaks are still in roughly the same places

Analysis of the time-resolved X-ray data was carried out using the programs FibreFix version 1.3 (Rajkumar et al. 2007) and Peakfit version 4 from AISN Software Inc. (Florence, OR, USA). For details see Eakins et al. (2016). The counts recorded by the X-ray detector for each pixel in the pattern are proportional to the intensity of the X-rays incident on that pixel. Diffraction patterns were aligned, background subtracted, quadrant-folded, converted to reciprocal space and equivalent frames from successive experiments were added to give the final summed patterns with good counting statistics. Because the myosin part of the pattern is well sampled and appears as sharp peaks it is very different from the axially smeared actin pattern which is continuous and weak. Our results on the myosin peak intensities are little affected by the actin pattern. For example, the backgrounds in the resting and active 1st layer line profiles in Fig. 2a are almost identical.

Modelling of diffraction patterns was carried out using Fiji image analysis software (Schindelin et al. 2012) and the HELIX program (Knupp and Squire 2004).

## Results

### Changes in the ML1 to ML3 layer line intensities and their interpretation

In diffraction patterns from resting vertebrate muscle, the relatively well ordered cross-bridges on the myosin filament surface give rise to a set of layer lines (ML1, ML2 etc.) which are orders of 43 nm (Huxley and Brown 1967; Squire 1972, 1981; Harford and Squire 1986, 1997; Squire and Knupp 2005; Hudson et al. 1997; Al-Khayat and Squire 2006). In patterns from bony fish muscle these layer lines are sampled by vertical row-lines at the same radial positions as the equatorial reflections, indicating the presence of the simple lattice of myosin filaments (Harford and Squire 1986). Similar patterns from frog muscles (Huxley and Brown 1967) also show sampling, but it is from a disordered and larger statistical superlattice unit cell and therefore much broader, less clear cut and more difficult to analyse than the fish muscle patterns (see Figs. 7.26 and 7.27 pp. 315/6 in Squire 1981-now reprinted).

What is evident from Fig. 2a is that the ML1 layer line is still sampled on the same row-lines as in relaxed muscle, showing that the simple lattice is still present and not greatly disordered. Similar sampling occurs on the higher order layer lines (ML2 out to ML6; see Fig. 1b). The equator of the diffraction pattern is still well-sampled in patterns from active muscle (Eakins et al. 2016), showing that the myosin filament lattice is still good. However, as detailed in the Discussion, a kind of disordering of the A-band structure that has previously been used to explain the broadening of the row-line sampling along the layer lines (Huxley et al. 1982) was claimed to be that caused by neighbouring myosin filaments getting out of axial register. The sampling on the equator would be unaffected by this, since it corresponds to what is seen in a view down the filament axis which would not be affected by axial displacements of the myosin filaments.

The fact that the ML1 to ML3 layer lines, off the meridian, still remain sampled in the active pattern demonstrates that any lattice disordering must not be very large. Later, in the Discussion Section, we show that the origin of the M3 broadening is, in fact, much more complicated than suggested by Huxley et al. (1982).

The fact that the ML1 to ML3 layer lines in patterns from active muscle are generally (not always) weaker than in patterns from relaxed muscle (e.g. Fig. 2a for ML1) has been taken in the past to imply that myosin heads have moved off the resting helix and have become disordered due to their interaction with actin (e.g. Huxley and Brown 1967). The fact that the layer lines do not totally disappear (Fig. 2) could mean one of two things. It could mean that some fibres in the whole fish muscle preparations have not been activated and are still in the original relaxed state. If this was the case then the remnant layer lines would just be reduced versions of the original layer lines and the intensities along each of the myosin layer lines would all reduce by exactly the same ratio. The alternative to this is that all the fibres in the muscle are fully activated and that the remnant myosin layer lines are, in fact, showing the presence of a different myosin head organisation in active muscle.

**Fig. 3:**
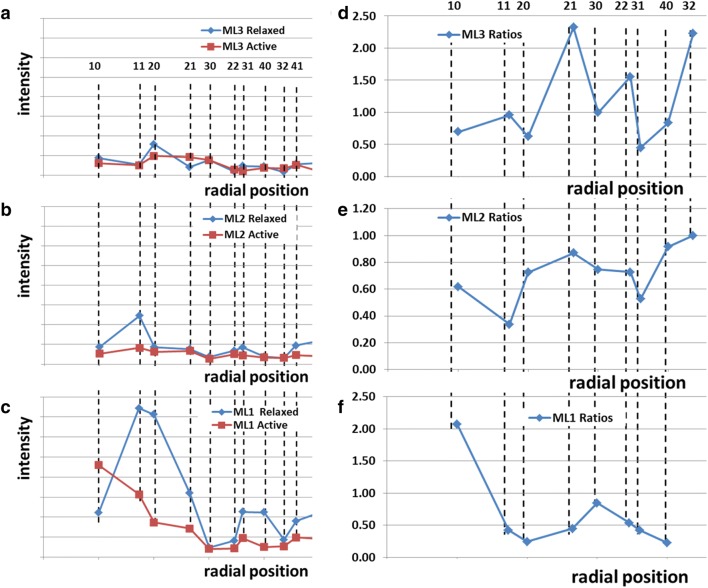
**a** Traces of the fitted intensities along the first three myosin layer lines (ML1, ML2 & ML3) from both resting (diamonds) and active (tetanus plateau: squares) diffraction patterns from bony fish muscle. Integration limits were: layer line 1: 0.0143 to 0.0306 nm^−1^; layer line 2: 0.0374 to 0.0536 nm^−1^; layer line 3: 0.06173 to 0.0779 nm^−1^. **d**–**f** Plots of the ratios (active/resting) of the peak intensities on the first three myosin layer lines. If the active intensities were just a weaker version of the resting pattern, as might occur if there were some fibres in the muscle that had not been activated, then the intensity ratios would all lie along horizontal lines. They do not do this, showing that the active layer lines are from a different crossbridge configuration from that in resting muscle. Numbers above the dashed lines indicate the row-line indices *h* and *k*

We tested these two possibilities by directly comparing the intensity profiles. The intensity profiles along each layer line were stripped out and the peaks fitted using Peakfit. The observed intensities from resting and active patterns were then plotted against radial position to see how they compared. The results are shown in Fig. 3a–c. It is evident that the active pattern is not just a scaled down version of the resting pattern. To make this more obvious we plotted the ratios of the intensities (active/resting) and these are shown in Fig. 3d–f. If the active pattern was just a reduced version of the resting pattern then these ratios should all lie along horizontal straight lines. This is not the case, which demonstrates that there is a different, myosin-centred, crossbridge arrangement in active muscle.

### Changes of the M3 meridional peak and its immediate off-meridional region

Previous interpretations of the M3 broadening on activation have been in terms of the myosin filaments in the A-band becoming axially disordered. As mentioned above, this would not affect the equator of the diffraction pattern (Eakins et al. 2016), but it was claimed to broaden the meridionals and off-meridional peaks. This possibility is analysed in the Discussion section. Clearly the ML1 to ML3 layer lines in Figs. 1, 2 and 3 remain sampled, perhaps with a slightly increased degree of disorder. But what happens to the M3 meridional peak?

**Fig. 4:**
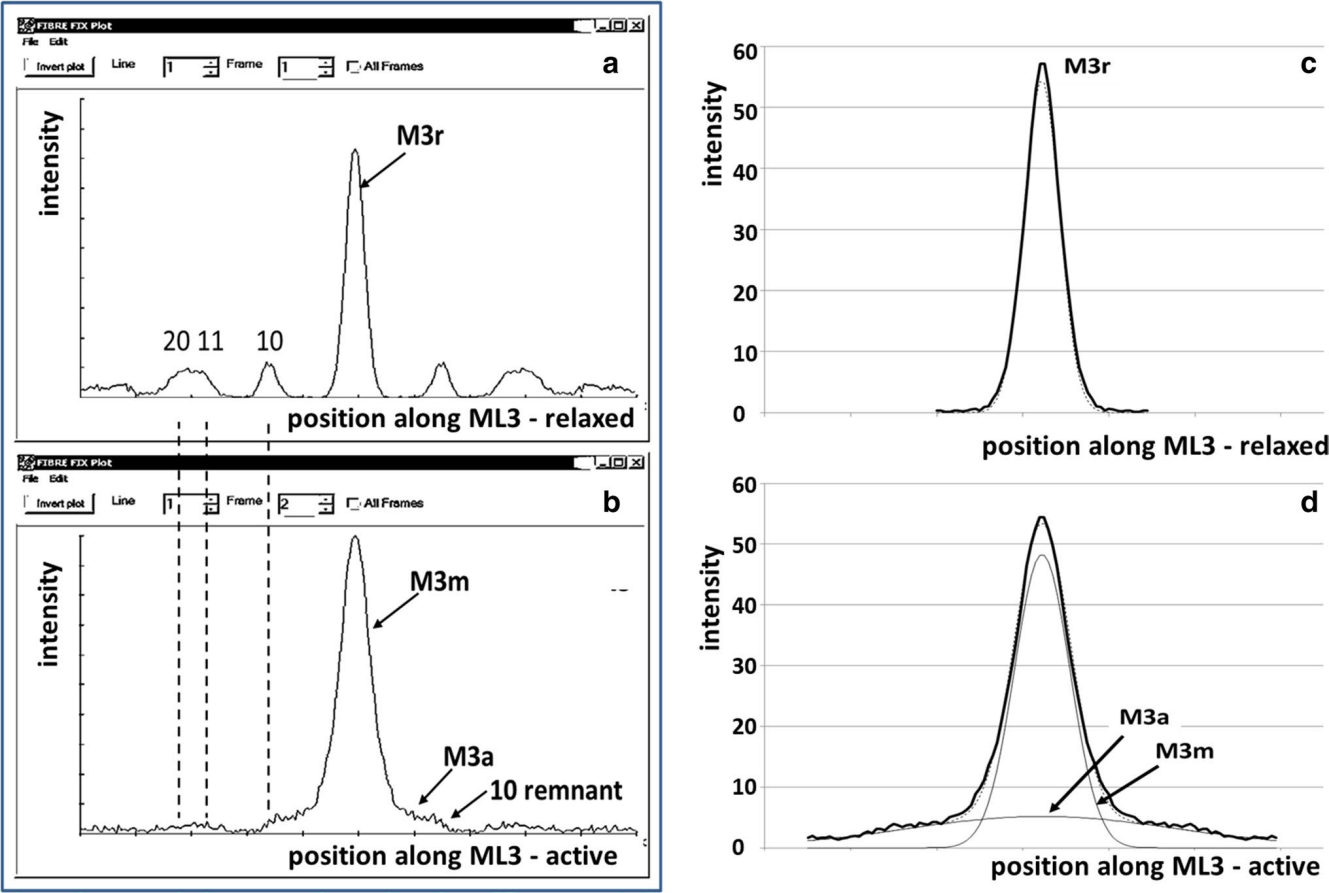
Intensity profiles across the meridian of the third myosin layer line at 14.3 nm spacing from **a** resting bony fish muscle (cf. Fig. 1) and **b** fully active muscle. The M3 position, labelled here as M3r (r for relaxed) and sampling along the layer line are indicated. **b** Shows the presence in active muscle of only a slightly broadened central M3 peak (marked M3m) and a new, shallow, but broad, peak labelled M3a. The remnant of the 10 row-line on the edge of the M3a peak is also shown. The images are shown as they appear in the FibreFix window apart from the labelling. Vertical scale is intensity, horizontal scale is position along the layer line. In **c**, **d** the peaks in **a**, **b** have been fitted with Gaussian profiles using in-house software. The resting M3 peak (M3r) can be fitted well with a single Gaussian function. The fitted active profile in **d** shows a similar, relatively sharp, meridional peak (M3m) superimposed on a much broader peak (M3a) which is also well fitted by a Gaussian

Figure 4 shows that in patterns from active bony fish muscle the main M3 peak (labelled M3m) is present, is only slightly broader than the resting M3r, and is similar in peak height to the peak from relaxed muscle. Note that the intensity scales in Fig. 4c, d are the same. In addition, the 10 peak on the third layer is much reduced. What is new is that superimposed on the M3m peak is a very broad, relatively shallow, peak labelled M3a.

Fitting of the peaks on the ML3 layer line from resting and active bony fish muscle (Fig. 4c, d), using in-house software, provided information both on the intensities and the nature of the peaks. For example, the new peak labelled M3a in the pattern from active muscle (Fig. 4d) is a peak that is centred on the meridian and is well fitted by a broad Gaussian function. Details of the peak fitting results are given in Table 1.

**Table 1:** Fitting of the inner ML3 peaks using PeakFit and in-house software

Row-line	Relaxed			Active		
	Position (pixels)	Peak height	Width (FWHM pixels)	Position (pixels)	Peak height	Width (FWHM pixels)
Meridian	0	54.52	9.7	0	48.34	15.2
New M3a				0	5.17	74.64
10	38.5	4.34	9.13	41.68	1.096	24.9
11	66.69	2.21	11.16			
20	77	4.25	17.37			
21	101.9	1.55	12.25			

Positions and widths are in pixel numbers. Widths and areas are raw ‘uncorrected’ values. See text for corrections

## Discussion

In summary, layer lines ML1 to ML3 in diffraction patterns from bony fish muscle together with the meridional M3 peak show that the simple lattice order is reasonably well maintained in active bony fish muscle. In addition, an extra meridional peak (M3a) appears at the M3 position in patterns from active muscle and the remnant of the ML1 to ML3 layer lines show a different intensity distribution from that from relaxed muscle, indicating a population of myosin heads in a different ‘active’ configuration. Here we consider what these new observations might mean.

As mentioned above, if meridional peaks like the M3 become broader across the meridian, but the equatorial peaks remain sharp, it has been claimed before (Huxley et al. 1982) that the origin of this is that the objects doing the diffracting, in this case the myosin filaments or myosin heads on actin filaments, have become axially disordered (i.e. shifted up and down along the fibre axis). We show below that, in fact, this is not the case. By modelling we show that, as the amount of axial disordering increases, the M3 intensity reduces, but the peak remains just as sharp across the meridian. Only when there is total axial disorder does the sampling disappear to leave the unsampled myosin filament or head-labelled actin filament diffraction pattern, which contains a very broad meridional peak on the third layer line. In the next section we discuss the various types of A-band disorder that might occur, and their expected effects on the diffraction pattern.

### Estimation of filament disorder

**Fig. 5:**
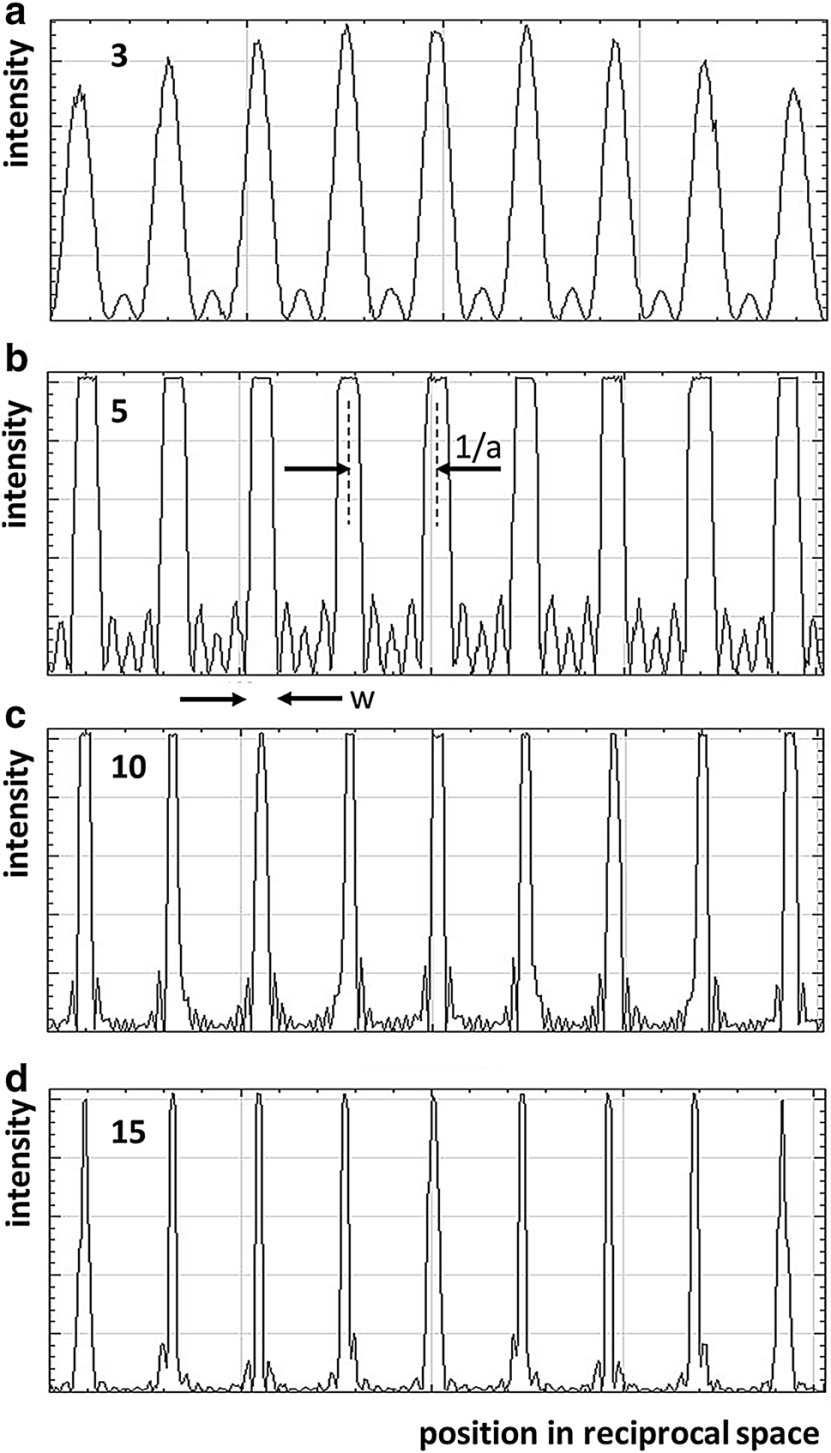
**a**–**d** Intensity profiles (lower parts) of the computed diffraction patterns from one-dimensional arrays of dots in arrays of varying length, but with the same repeat (separation between dots) ‘a’. (Intensity vertical against position in the diffraction pattern horizontal). The number of repeats from the top is **a** 3, **b** 5, **c** 10 and **d** 15. It can be seen that, as the array size increases, the width w of the peaks (here full width at the peak base—see arrows in plot in **b** as an example) reduces systematically. In other words, the extent of the array can be determined from the width of the peaks. Patterns were generated using the HELIX program (Knupp and Squire 2004) and plots were created in Fiji

**Fig. 6:**
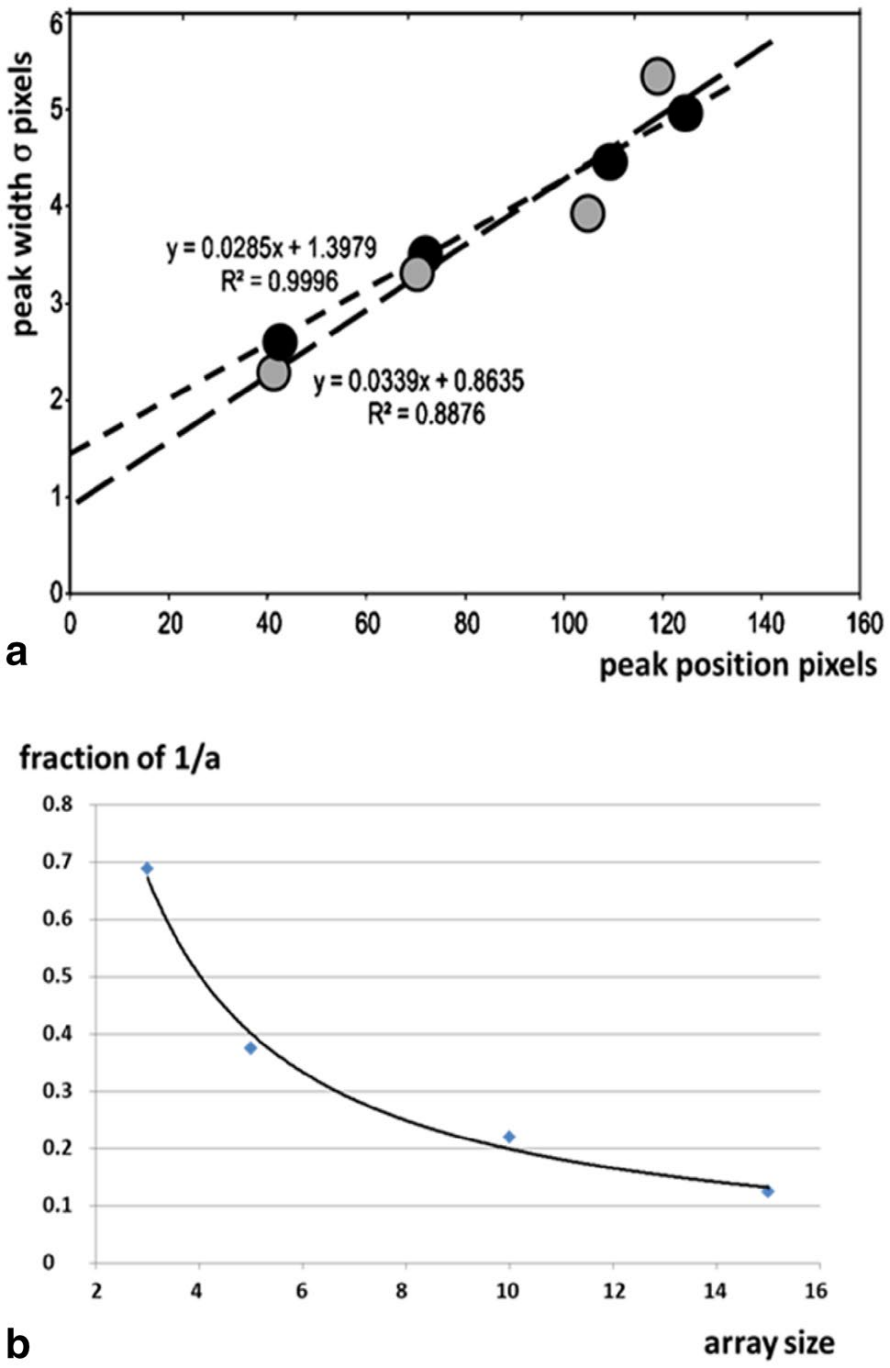
**a** Plots of peak width against radial position along the equator (see Fig. 2b) for resting muscle (grey symbols, long dashes) and active muscle (black symbols, short dashes). In these cases the plots are almost linearly related, indicating only very slight paracrystalline disorder. The intercept at the origin reveals the beam width ($\varvec\sigma_{\text{c}}$) convoluted with the array size width ($\varvec\sigma_{\text{p}}$). The plots are very similar for resting and active muscle suggesting that the lateral extent of the coherent unit of myosin filaments is similar in both cases, as judged by the equatorial peaks. **b** Variation of the full width of the main peaks from arrays of different extents in Fig. 5a–d as a fraction of the 1/a spacing of the reciprocal lattice. This gives information about $\varvec\sigma_{\text{p}}$ in Eq. 1

To analyse the details of the disorder in the lattice, the observed Gaussian widths of the equatorial peaks can be plotted as a function of position along the equator, as in Fig. 6a. As detailed in Yu et al. (1985), and amplified here by adding an additional term, the peak width ${\varvec{\sigma}}_{hk}$ would be expected to be of the form:

1 $${\varvec{\sigma}}^{{\mathbf{2}}}_{{\varvec{hk}{\mathbf{(obs)}}}} = {\varvec{\sigma}}^{{\mathbf{2}}}_{{\mathbf{c}}} + {\varvec{\sigma}}^{{\mathbf{2}}}_{{\mathbf{p}}} { + }({\varvec{\sigma}}^{{\mathbf{d}}}_{{\varvec{hk}}} )^{{\mathbf{2}}} + ({\varvec{\sigma}}^{{\mathbf{s}}}_{{\varvec{hk}}} )^{{\mathbf{2}}}$$

where ${\varvec{\sigma}}_{\text{c}}$ is the width of the direct beam, ${\varvec{\sigma}}_{\text{p}}$ is the intrinsic broadening due to the finite array size (i.e. particle size) of the A-band lattice, even if perfectly ordered within that lattice (see Fig. 5a–d); it is the same for all ***h***,***k***), ${\varvec{\sigma}}^{{\mathbf{d}}}_{{\varvec{hk}}}$ is broadening due to the distribution of inter-filament spacings and ${\varvec{\sigma}}^{{\mathbf{S}}}_{{\varvec{hk}}}$ is the broadening due to any lateral paracrystalline disorder in the array of filaments (how much deviation occurs away from straight lattice planes). The third and fourth terms on the right of Eq. 1 disappear on the meridian (Miller indices ***h*** and ***k*** are both zero), but the effect of the beam size and the extent of the array both affect the peak width across the meridian.

We are dealing with Gaussian fits to the layer line data, where the bell-shaped Gaussian Function **g(x)** has the form:

$${\mathbf{g}}\left( {\mathbf{x}} \right) = \, [a/{\varvec{\sigma}}({\mathbf{2\pi }})^{\raise.5ex\hbox{$\scriptstyle 1$}\kern-.1em/ \kern-.15em\lower.25ex\hbox{$\scriptstyle 2$} } ] \, {\mathbf{exp}} \, \{ - \raise.5ex\hbox{$\scriptstyle 1$}\kern-.1em/ \kern-.15em\lower.25ex\hbox{$\scriptstyle 2$} (\left( {{\mathbf{x}} \, {-}b} \right)/{\varvec{\sigma}})^{{\mathbf{2}}} \}$$

and ***a*** is the area, ***b*** is the position of the centre of the peak and ${\varvec{\sigma}}$ can be thought of as controlling the width of the “bell”.

The Full Width at Half Maximum (FWHM) value of any peak is obtained from:

2 $${\mathbf{FWHM}} \, = \, {\mathbf{2}}{\mathbf{.35482}}\;{\varvec{\sigma}}$$

The size of the peaks in the observed diffraction pattern is determined by not only the beam size, but also the intrinsic broadening due to the extent of the lattice and the disorder as in Fig. 6. If the main beam ($\varvec\sigma_{\text{beam}}$) and the intrinsic broadening due to the A-band lattice character ($\varvec\sigma_{\text{struct}}$) are both taken as Gaussian functions, and these are convoluted together to give the observed peaks, which would then also be Gaussian in form as observed, then for the width of the M3 peak we can use the relationship:

3 $${\varvec{\sigma}}^{{\mathbf{2}}}_{{{\mathbf{obs}}}} = {\varvec{\sigma}}^{{\mathbf{2}}}_{{{\mathbf{beam}}}} + {\varvec{\sigma}}^{{\mathbf{2}}}_{{{\mathbf{struct}}}}$$

where ${\varvec{\sigma}}_{\text{obs}}$ is the observed peak width, ${\varvec{\sigma}}_{\text{beam}}$ is the part of ${\varvec{\sigma}}_{hk}$ due to beam size and ${\varvec{\sigma}}_{\text{struct}}$ is the broadening due to lattice disorder of any kind that is not a function of ***h*** and ***k***. In other words, in the case of the M3 peak, on the meridian of the diffraction pattern, the 3rd and 4th terms in Eq. (1) do not apply (*h* and *k* are both zero) and we are only dealing with the effects of the beam size and the extent of the lattice, together with anything else that might affect the peak width. Note that a lack of parallel alignment of fibres or myofibrils within the muscle would also cause arcing of the reflections and broadening of the meridional peaks across the meridian. However, it would also cause fanning of the layer lines and there is very little evidence for this in the diffraction patterns in Fig. 1. Disorientation appears to be a minor contributor to what is seen.

The ${\varvec{\sigma}}$ values for the equatorial peak width plots from resting and active muscle from the intercept at the zero pixel position (Fig. 6a) are 0.8635 and 1.3979 respectively. Since the beam itself has not changed between the resting and active patterns, and if the lateral extent of the coherent unit only changes by a small amount, then we can take the average of the two intercepts as the best estimate of the FWHM due to the main beam and array size **(**${\varvec{\sigma}}_{\text{beam}}$**)** as 2.35482 × (0.8635 + 1.3979)/2 = 2.6626 pixels. The beam itself is about 1.3 pixels wide, so the broadening due to the lattice extent is ${\varvec{\sigma}}_{\text{struct}}$ = 2.3 pixels (giving an estimated full width of 2.3 × 1.82 = 4.19 pixels). The 10 row-line is found at 38.5 pixels (Table 1), so width fraction of 1/a is 0.11 and from Fig. 6b the lattice extent would be about 20 unit cells. The equatorial peaks are always sharper than the layer lines, so this large lattice extent does not apply when the A-band structure is considered in 3D. For example small rotations of the myosin crowns around the filament axis will limit the simple lattice sampling of the myosin layer lines, but the equator would be little affected. (Note that the factor 1.82 is the ratio between the full width at one-tenth maximum and the full width at half maximum of a Gaussian. We are using full width at one tenth maximum as an estimate of the full width of the peaks).

### The effects of axial misalignment of the myosin crowns

**Fig. 7:**
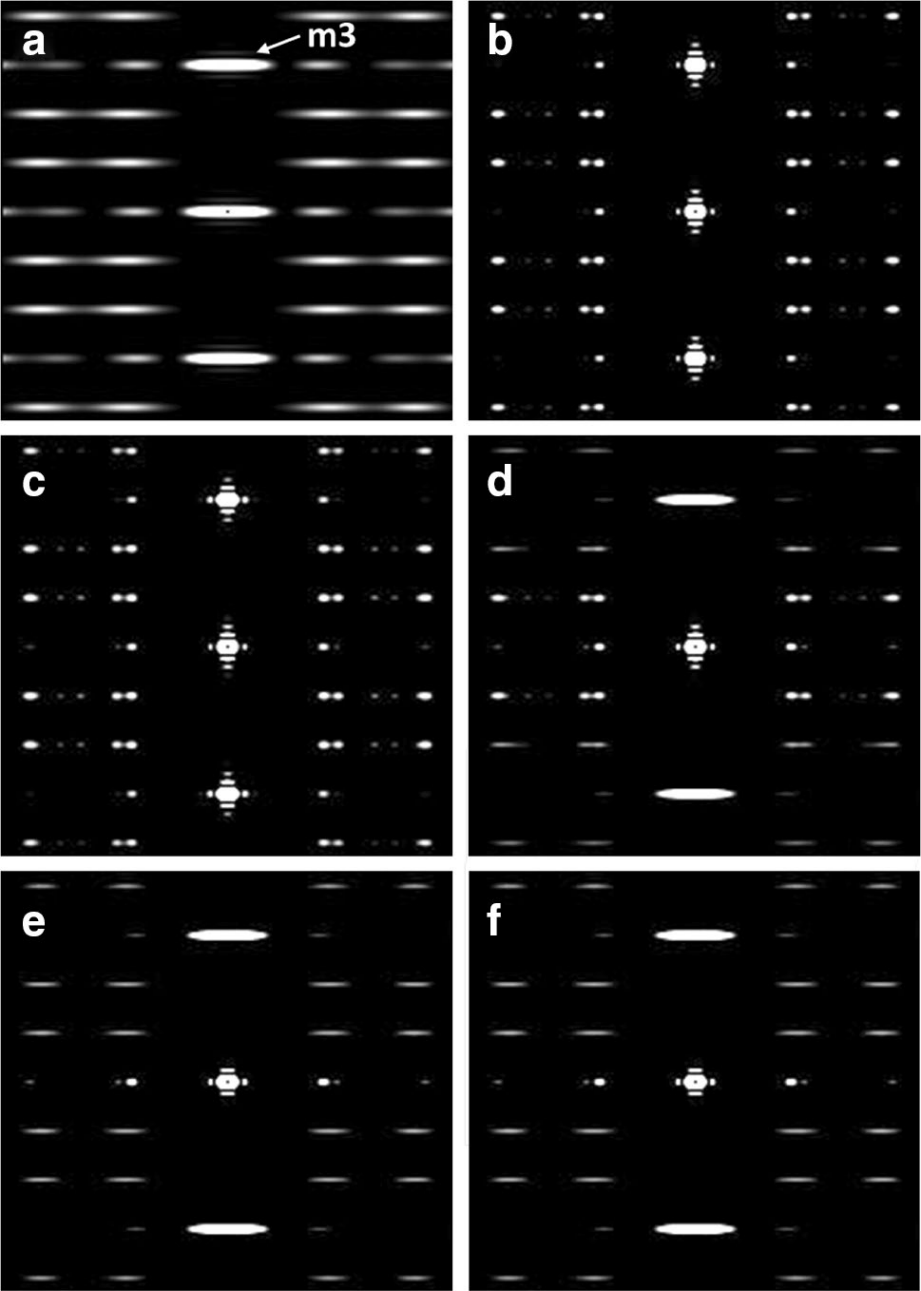
**a** Computed diffraction pattern from a single, simulated, myosin filament model with spheres placed at 15 nm radius on a 3-start 9/1 helix of subunit axial translation (inter-crown spacing) of 14.3 nm. **b**–**f** Computed diffraction patterns from an array of myosin filaments as in **a** and of lateral extent 100 nm, but with random axial shifts within Gaussian distributions of different widths along the fibre axis. Each pattern is the average of patterns from 100 distributions of filaments. Axial shifts widths were: **b**${\varvec{\sigma}}_{\text{a}}$ = 0, **c**${\varvec{\sigma}}_{\text{a}}$ = 1 nm, **d**${\varvec{\sigma}}_{\text{a}}$ = 5 nm, **e**${\varvec{\sigma}}_{\text{a}}$ = 10 nm and **d**${\varvec{\sigma}}_{\text{a}}$ = 20 nm. The M3 peak is indicated in **a**. Patterns were computed using a modified version of Helix (Knupp and Squire 2004)

In order to test the possible effects of axial misalignment of the myosin filaments, we set up a series of models and calculated their diffraction patterns. The models contained 25 ‘myosin filaments’ in a hexagonal array. Since we are looking at effects on the M3 in patterns from active muscle, we do not yet know what the ordered ‘active’ crossbridge arrangement on each myosin filament is like, so, to test the effects of axial misalignment on a model system, we simulated a schematic myosin filament by putting spheres at a radius of 15 nm from the filament axis and arrayed on the 3-stranded 9/1 helix of vertebrate muscle myosin filaments (Squire 1972). We then applied random axial shifts to these filaments with the axial displacements randomly chosen within a Gaussian distribution of axial positions defined by an axial spread ${\varvec{\sigma}}_{\text{a}}$ of the Gaussian. The diffraction pattern from this array was then computed. For each value of ${\varvec{\sigma}}_{\text{a}}$ the filament distribution was calculated 100 times and all 100 diffraction patterns were added together to give the final pattern (i.e. the ‘average’ pattern for about 2500 filaments). The results are illustrated in Fig. 7, starting from a single filament and its diffraction pattern (a), and then showing the effects of gradually increasing ${\varvec{\sigma}}_{\text{a}}$ values.

The conclusion from the results in Fig. 7 is that the width across the meridian does not change appreciably as the axial disorder gets worse from (b) through to (c), but after that the sampling of the ML2, ML3 layer lines and higher orders disappears and in (f) the sampling of ML1 has also gone, leaving behind the unsampled myosin filament diffraction pattern as in (a). At the same time, when the row-line sampling can be seen, the lateral width is largely unaltered as ${\varvec{\sigma}}_{\text{a}}$ increases, As expected, sampling on the equator remains the same throughout.

**Fig. 8:**
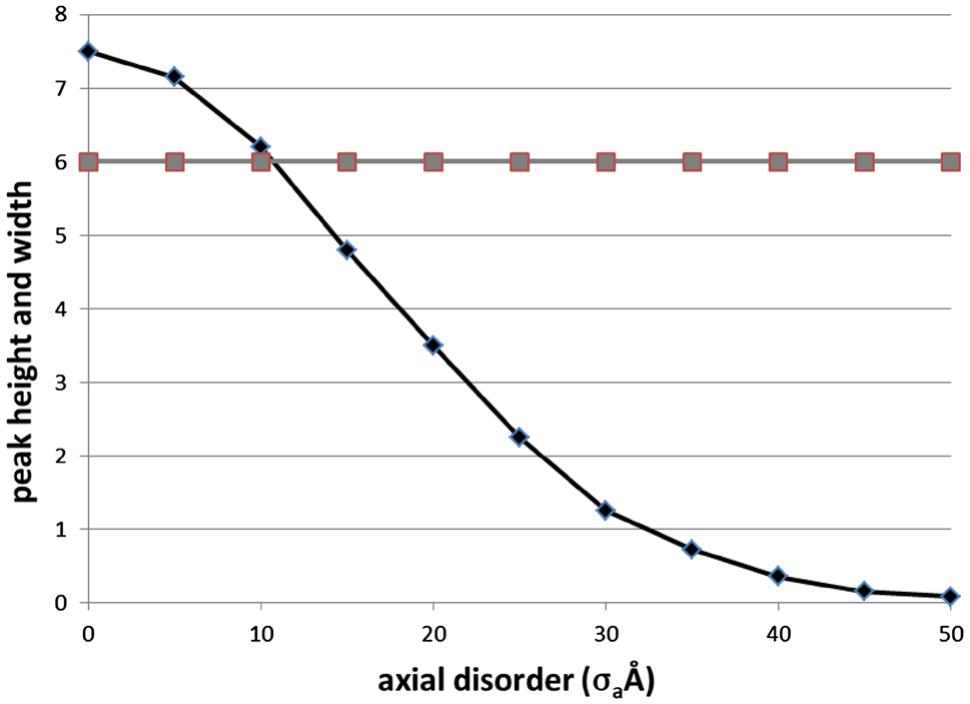
Plots of the M3m peak height (black line, diamonds, arbitrary scale) and peak width ${\varvec{\sigma}}_{\text{a}}$ (grey line, square symbols, pixels) from calculations similar to those illustrated in Fig. 7. As the axial disorder increases the peak height of the M3m drops systematically, but the peak width remains constant

Figure 8 shows plots of the calculated M3 width and peak height as a function of the amount of axial disorder in a system such as that in Fig. 7. The key point here is that, although the M3 peak height drops dramatically as ${\varvec{\sigma}}_{\text{a}}$ increases, the width of the peak does not change at all. The width of the peak is solely determined by the beam size and the extent of the A-band lattice and, contrary to the suggestions of Huxley et al. (1982), not by any axial disordering. The final effect of very high axial disordering is simply to remove the sampling completely and to restore the unsampled myosin filament diffraction pattern (Fig. 7f). The central, meridional, part of the unsampled filament diffraction pattern at M3 is, of course, much broader than the sampling peaks if ${\varvec{\sigma}}_{\text{a}}$ is not large enough to cut out the row-line sampling.

**Fig. 9:**
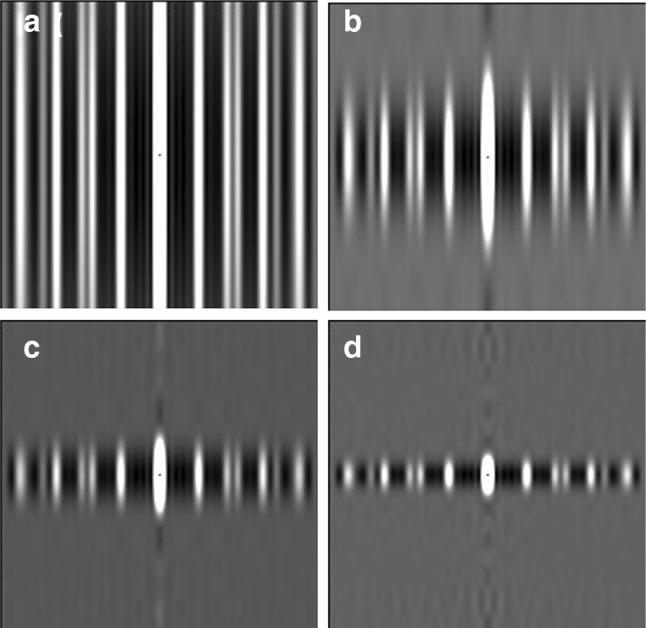
Changes in the row-line interference function as the axial extent of the myosin filament disorder gradually increases, with ${\varvec{\sigma}}_{\text{a}}$ equal to **a** 1 nm, **b** 5 nm, **c** 10 nm, **d** 20 nm. These are the functions that would sample the myosin layer-lines for different values of ${\varvec{\sigma}}_{\text{a}}$

To complete this discussion, Fig. 9 shows the form of the row-line interference function by which the layer lines would be sampled as the axial disorder parameter ${\varvec{\sigma}}_{\text{a}}$ changes from (a) 1 nm, to (b) 5 nm, (c) 10 nm and (d) 20 nm. Laterally the row-lines do not change at all, but their axial extent gradually reduces as ${\varvec{\sigma}}_{\text{a}}$ increases.

### Estimation of the A-band coherent lattice size for the myosin crowns

The limit to the extent of the A-band myosin filament crown lattice in the bony fish muscle specimen used here, both relaxed and active, can be determined from the observations in Table 1. Considering now the main M3 peaks from resting and active muscle (Fig. 6: M3r and M3m), the full widths at half maximum (FWHM) of the observed peak profiles are 9.7 pixels for resting muscle and 15.16 pixels for active muscle. Allowing for the beam width and array size, as determined from the equatorial peaks (Fig. 6a), reduces these figures to 7.4 and 13.8 pixels respectively for the FWHM. The ‘**a**’ spacing is 44.95 nm, so the 10 spacing is d = 0.866 × 44.95 = 38.93 nm, and 1/d for the 10 row line is 0.0257 nm^−1^. The 10 row-line is found at 38.5 pixels (Table 1), so the M3r and M3m FWHM values of 7.8 and 13.8 pixels correspond to 0.0052 and 0.0092 nm^−1^ respectively.

Referring to Fig. 6b, the fractions of the 1/a spacings are 0.20 × 1.82 = 0.364 and 0.36 × 1.82 = 0.655, corresponding to 6 and 4 unit cells respectively. As above, the factor 1.82 is the ratio between the full width at one-tenth maximum and the FWHM for a Gaussian.

### Evidence for two different crossbridge arrangements in active muscle

We have shown from the irregular ratios of the active sampled layer line peaks to the resting peaks (Fig. 4) that in active muscle there is a different crossbridge arrangement centred on the myosin filaments. We know that this new arrangement is still centred on the myosin filaments because the pattern from the actin filaments, the usual actin layer-lines based on an axial repeat of around 36 nm, is not sampled, but has broad smoothly varying intensities along the layer lines, indicating considerably more disorder of the actin filaments compared to the myosin filaments.

**Fig. 10:**
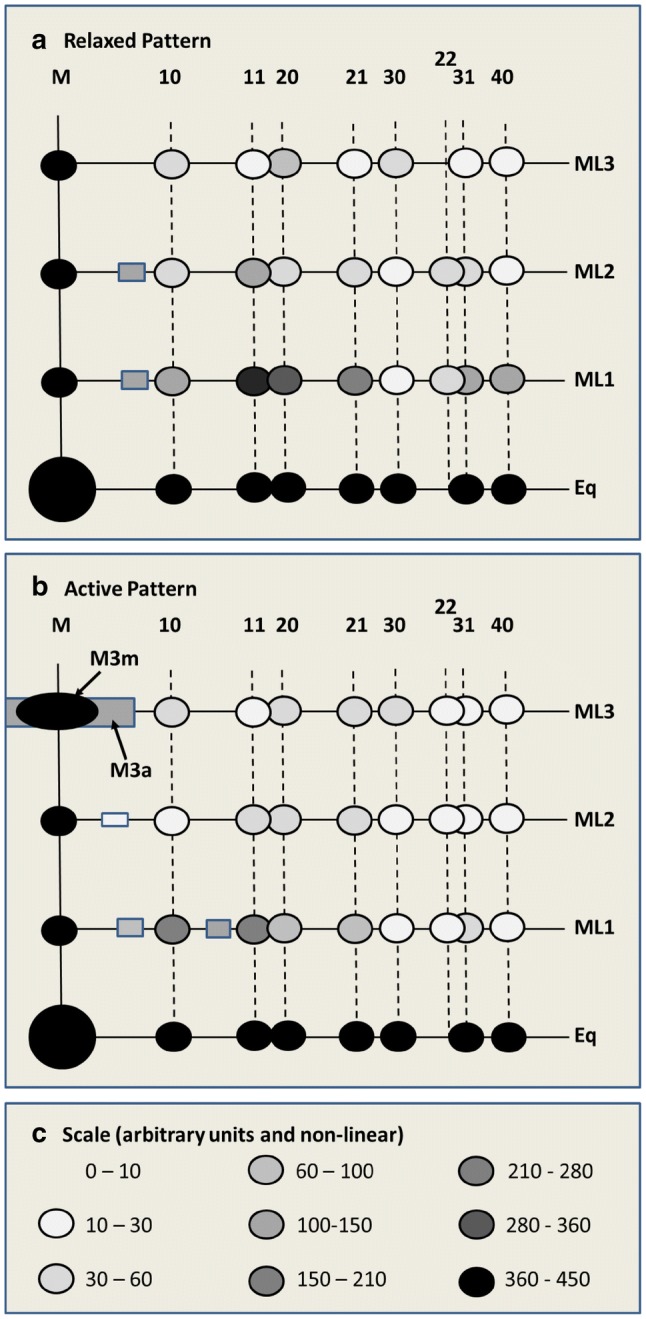
Schematic visual summaries of the different observed diffraction patterns recorded from fish muscle in the contractile cycle: **a** relaxed pattern between tetani, **b** active pattern at the plateau of the tetanus, **c** intensity scale (non-linear). The meridian and equator are strong and have just been included as black dots for completeness. Note that in all patterns there are some weak peaks that do not lie on the expected row-lines. These are shown as shaded rectangles. The only strong peak that does not lie on a row-line is the M3a peak which is completely absent in patterns from relaxed muscle (**a**), but significant in patterns from active muscle (**b**), where it is a broad meridional peak underneath the usual M3 peak (M3m). The pattern in **b** is a mixture of the patterns from active states 1 and 2

So, from the myosin layer line data, we have three known states. One is the normal resting structure (Hudson et al. 1997), the second is this new active myosin-centred arrangement which we term ‘active state 1’ which shows all the characteristics of being from the myosin filament lattice. The third is the arrangement of heads that gives rise to the M3a peak (Figs. 5, 6) on the ML3 layer line. The M3a peak does not have the characteristics of the row-lines from the myosin filament lattice; it is a very much broader peak than the M3m peak, and since the rest of the ML3 layer line is still sampled by the lattice it does not appear to come from the same pattern as the sampled peaks. We term the crossbridge state associated with the M3a peak ‘active state 2’. The diffraction patterns from these three configurations are simplified and summarised in Fig. 10.

Can we identify what active states 1 and 2 are? The two obvious contenders are: (i) the weak-binding state or early pre-powerstroke states which bind actin, but not in a stereospecific way, and (ii) the strong states with their motor domains stereospecifically-attached to actin and the lever arms presumably at a variety of different angular positions.

The resting pattern from bony fish muscle has already been modelled by Hudson et al. (1997), Al-Khayat and Squire (2006). This was achieved by describing the myosin crossbridge arrangement in resting muscle in terms of such parameters as origin radius, head tilt, head slew, head rotation, angles between the motor domain and lever arm and so on, and then searching over parameter space using a simulated annealing procedure to get the best fit to the observed diffraction pattern. Assessing the goodness of fit using an R-factor gave a good and sensible structure, which we discuss further elsewhere (Knupp et al. 2019).

The only known active crossbridge state that is still myosin-centred, in the sense that the heads stay with their lever arms pointing back towards their origins on the myosin filaments (see Fig. 12a in Eakins et al. 2016), is the weak binding state. The outer parts of these heads would be binding transiently to actin, but not stereospecifically, so they do not conform to the symmetry of the actin filament; they are therefore not actin-centred, but are still myosin-centred. For these reasons active state 1 heads may well be the weak-binding/pre-powerstroke heads. In principle, exactly the same procedure can be carried out to model this sampled part of the diffraction pattern from active muscle (M3m;  i.e. active state 1) as was done for the resting pattern (Hudson et al. 1997). But, if it really is describing the weak-binding/pre-powerstroke bridges, then there would be need to be additional parameters to fit to allow for the different possible azimuthal shifts of the heads to make them point towards the actin filaments.

The third structure is whatever gives rise to the M3a peak on the ML3 layer line. If this peak does not come from the myosin filament array, and we have argued above why we think this is so, what is there in the muscle that could give rise to it? We know that, whatever structure it is, it cannot be myosin-centred because it does not have the same peak width as the other myosin-centred peaks on the meridian and on the simple lattice row-lines. Secondly, it must be due to myosin heads because it has the characteristic 14.3 nm myosin crossbridge axial repeat. We know that myosin heads attached to actin can still show a 14.3 nm axial repeat, even though there are no actin monomers at this axial separation, because rigor muscle also shows a 14.3 nm meridional peak even though all the myosin heads bind to actin (Cooke and Franks 1980; Lovell et al. 1981; Squire and Harford 1988; Yagi 1996). What is seen is the average labelling from a mixture of crossbridge separations along the actin long-pitched helices which are 2 × 5.54 = 11.8 nm apart (assuming a 13/6 actin helix of repeat 36 nm) or 3 × 5.54 = 16.62 nm along the same long-pitched strand. The axial separation of actin-bound heads in opposite strands could be 13.85 (± 5.54) nm. Also, the actin filaments in bony fish muscle are not well enough ordered for their layer-lines to be sampled by the simple lattice, so peaks that are broad across the meridian and along the layer lines would be expected.

The M3a full width at half maximum (FWHM) is 74.64 pixels (Table 1), relative to the main M3m peak from active muscle which is only 13.8 pixels wide. This new M3a peak is entirely consistent with it being from myosin heads attached to actin in a strong binding state and showing the disorder of the actin filaments. Only in strong states will the myosin heads be actin-centred, with their motor domains stereospecifically-attached to actin and therefore following the actin symmetry. The observed uncorrected width of the M3a peak of 74.64 pixels, after correction for the beam width, becomes a FWHM value of 0.0013 Å^−1^ for the muscle at 2.3 µm sarcomere length. For comparison, the (FWHM) widths of the M3 peaks in diffraction patterns from rigor fish muscles at 2.2 and 2.5 µm sarcomere lengths (Eakins et al. 2018) were 0.00082 and 0.0012 Å^−1^ respectively, remarkably close to that of the M3a peak. So the ordered part of the observed active M3 peak from bony fish muscle (M3m) probably comes from the myosin-centred weak-binding and pre-powerstroke heads and the broad M3a meridional peak probably comes from actin-attached heads in the actin-centred strong states.

With these myosin head states tentatively identified, how can we follow the progress of the myosin heads through the whole contractile cycle; how can we produce ‘Muscle—the Movie’?

### How to tackle ‘Muscle—the Movie’

The whole time-resolved, 2D, low-angle X-ray diffraction pattern from active bony fish muscle out to about 6 nm resolution has already been recorded using the Daresbury synchrotron and the equator of this pattern has already been analysed (Eakins et al. 2016). The strongest parts of the diffraction pattern are the meridian and equator and these parts of the pattern can be recorded with quite good counting statistics. The off-meridional parts of the ML1 to ML6 layer lines are relatively weak and it is therefore harder to get good time-resolved data for them. The original timing protocol recorded the pattern for 100 ms at the resting phase prior to contraction, at 1 ms time intervals during the rapidly changing rising phase of the tetanus, for 100 ms at the tension plateau, and at 4 ms intervals on the relaxation phase (Eakins et al. 2016). The weak layer line peaks during the 1 ms time steps were therefore recorded with relatively poor counting statistics.

Despite these reservations, modelling such as that carried out by Hudson et al. (1997), Al-Khayat and Squire (2006) using the MOVIE program allowed an initial ‘resting’ structure to be determined based on the known symmetry and spacings of vertebrate myosin filaments, together with known molecular domain structures (e.g. the myosin heads). As described above, this was achieved by parameterising the positions of the moving domains, and then searching over these parameters using a simulated annealing process to optimise the fit between the observed and calculated myosin layer-line intensities. The models of Hudson et al. (1997) for fish muscle and of Al-Khayat et al. (2003) for insect flight muscle did not generate heads with the interacting heads motif (e.g. Al-Khayat et al. 2013; Hu et al. 2016). But we show elsewhere (Knupp et al. 2019) that the interacting heads motif structures for vertebrate and insect flight muscle myosin filaments do not, in fact, explain the observed resting X-ray diffraction patterns. The resting X-ray patterns that we are modelling come from a myosin head arrangement different from the interacting head motif. The earlier modelling of the X-ray diffraction data appears to have been reliable; a conclusion that is essential if we are to produce ‘Muscle—the Movie’.

In order to produce ‘Muscle—the Movie’ the whole A-band lattice structure needs to be included; myosin filaments, actin filaments (including troponin and tropomyosin), moving myosin heads (position and shape) and possibly C-protein (MyBP-C) too (e.g. Luther et al. 2011). So, for example, appropriate parameters can control the position, rotation, azimuthal angles and lever arm tilts of the myosin heads, and the relative positions and shapes of the actin globular domains, tropomyosin and troponin. MusLabel (Squire and Knupp 2004) can help to define which actin monomers are likely to be labelled with heads. Subsequently, the calculated diffraction patterns can then be compared to the experimental data by the computation of the crystallographic R-factor (goodness of fit factor). In principle, this can be done throughout the contractile cycle by modelling each timeframe of data and using not only the myosin layer lines reported in this paper, but using the actin layer-lines too. We showed in Eakins et al. (2018) how analysis of the actin layer lines can be very informative.

Looking at successive 1 ms time-frames through the rising phase of the tetanic contraction shows that each of the sampled peaks on the myosin layer lines changes only slowly from their initial relaxed values. These small changes can be used, for example, to compare the 2nd timeframe structure with the relaxed structure (the 1st frame) that has already been modelled by Hudson et al. (1997). To do this we can use Fourier difference synthesis. Fourier difference synthesis is a standard technique used to generate a density difference map based on two similar, but not identical, diffraction patterns and it shows where density has moved from and where it has moved to. In a normal X-ray diffraction pattern, each diffraction peak is associated with an intensity and a phase, but only the intensity is actually recorded. However, if the intensities can be modelled, as was done in Hudson et al. (1997) for vertebrate muscle and Al-Khayat et al. (2003) for insect flight muscle, then ‘model’ phases can be calculated. For resting muscle we have observed intensities (or amplitudes; amplitude is the square root of the intensity) and calculated ‘model’ phases. From a set of amplitudes and phases, the diffracting structure can be reconstructed in the computer using the process of Fourier synthesis. Going on to the 2nd frame, from which we know the intensities (hence amplitudes), but not the phases, the ‘model’ phases from frame 1 can be used with the frame 2 amplitudes to generate a new ‘hybrid’ reconstruction which contains more information about the structure giving frame 2. The density changes between the reconstructions from frame 1 and frame 2 show how the original frame 1 model needs to be changed. As mentioned above, such a density difference map can be computed directly by Fourier difference synthesis. So the positions of the moving parts of the contractile machinery can be followed incrementally through the cycle, with the structure for each time frame being modelled by Fourier difference synthesis based on the structure in the previous timeframe. The myosin head arrangement seen in active state 1 would be expected to be a milestone through this structural cycle and will help to guide the process, as will modelling of the strong states on actin.

In summary, in the past, two pieces of software have been developed to carry out this kind of modelling process. The MOVIE program allowed the diffraction patterns from resting fish and insect flight muscles to be solved (Hudson et al. 1997; Al-Khayat et al. 2003), and MusLabel (Squire and Knupp 2004) permitted the simulation of the way myosin heads might interact with actin for different sarcomere geometries. To produce the final Movie these two programs need to be merged into one, and the capability of modelling actin, tropomyosin and troponin also needs to be added. Because of the symmetry mismatch between the axial periodicities of the actin and myosin filaments (about 36 and 43 nm respectively, giving a beat period of around 5 × 43 = 6 × 35.8 = 215 nm), we predict that in active muscle every myosin head in the 215 nm long unit cell will behave slightly differently. MusLabel can calculate the probability of each head attaching to a particular actin monomer throughout this length and MOVIE can generate a 3-dimensional model of the A-band which takes into account the predictions from MusLabel as well as the steric constraints of the A-band and the different amounts of disorder in the actin and myosin filament arrays. Because of the stochastic nature of the predictions from MusLabel, several different configurations of the A-band unit cell can be created in parallel and an averaged diffraction pattern calculated from them to be compared to the experimental one. The whole 2D diffraction pattern can be computed and all these models can be varied independently until the best overall R-factor between the observed and calculated 2D patterns is obtained. This approach permits the natural structural variability of the A-band to be dealt with.

## Conclusion

Because of the enormous computing demands required for it, the process of producing ‘Muscle—the Movie’ will be very challenging, but in principle it can be done as described above. We have already solved the resting pattern from bony fish muscle and, as shown here, there is another pattern (active state 1; Fig. 10b), which we think is the weak-binding state, that can be solved in an analogous way. As discussed above, the challenging part will be to follow the crossbridge and other protein (actin, troponin etc.) positions during the rising phase of the tetanus, especially because of the shortness of the time slices (~ 1 ms) needed to do the job properly. But, with ever-increasing computing power, and with time-resolved experiments such as those in Eakins et al. (2016) being carried out on the latest synchrotron beam lines with their high intensity and fine focus, and using state of the art area detectors, the quality of the data should improve dramatically. It will then be possible to use the resting structure as the starting point and to see what small incremental changes in structure are needed to explain the small changes in intensity in the 2D diffraction pattern that occur in successive 1 ms timeframes. This task will be eased by the knowledge of the various components of the A-band, such as the myosin heads (Rayment et al. 1993; Dominguez et al. 1998), actin filament structure (Chou and Pollard 2019), the shape and location of troponin (Paul et al. 2017), the interactions of C-protein with actin (Luther et al. 2011) and the way heads bind to actin in rigor (Holmes et al. 2004; Behrmann et al. 2012; von der Ecken et al. 2016; Fujii and Namba 2017). In the case of the myosin layer lines, the use of Fourier difference synthesis techniques should show directly how the myosin head conformations need to change as the cycle progresses.
